# Nanotechnology-Assisted Biosensors for the Detection of Viral Nucleic Acids: An Overview

**DOI:** 10.3390/bios13020208

**Published:** 2023-01-30

**Authors:** Hye Kyu Choi, Jinho Yoon

**Affiliations:** 1 Department of Chemistry and Chemical Biology, Rutgers, The State University of New Jersey, Piscataway, NJ 08854, USA; 2 Department of Biomedical-Chemical Engineering, The Catholic University of Korea, Bucheon-si 14662, Gyeonggi-do, Republic of Korea

**Keywords:** biosensor, nanotechnology, viral nucleic acids, electrochemistry, Raman spectroscopy, fluorescence

## Abstract

The accurate and rapid diagnosis of viral diseases has garnered increasing attention in the field of biosensors. The development of highly sensitive, selective, and accessible biosensors is crucial for early disease detection and preventing mortality. However, developing biosensors optimized for viral disease diagnosis has several limitations, including the accurate detection of mutations. For decades, nanotechnology has been applied in numerous biological fields such as biosensors, bioelectronics, and regenerative medicine. Nanotechnology offers a promising strategy to address the current limitations of conventional viral nucleic acid-based biosensors. The implementation of nanotechnologies, such as functional nanomaterials, nanoplatform-fabrication techniques, and surface nanoengineering, to biosensors has not only improved the performance of biosensors but has also expanded the range of sensing targets. Therefore, a deep understanding of the combination of nanotechnologies and biosensors is required to prepare for sanitary emergencies such as the recent COVID-19 pandemic. In this review, we provide interdisciplinary information on nanotechnology-assisted biosensors. First, representative nanotechnologies for biosensors are discussed, after which this review summarizes various nanotechnology-assisted viral nucleic acid biosensors. Therefore, we expect that this review will provide a valuable basis for the development of novel viral nucleic acid biosensors.

## 1. Introduction

In recent decades, the diagnosis of fatal viral diseases has attracted increasing attention among researchers in the medical and biosensor fields, especially in the aftermath of recent disease outbreaks [1,2]. Particularly, since the beginning of 2020, the global spread of infectious viral diseases such as the severe acute respiratory syndrome coronavirus 2 (SARS-CoV-2) and monkeypox virus (MPXV) has claimed the lives of thousands of people worldwide [3,4,5]. SARS-CoV-2 is a respiratory virus that not only causes critical economic and social impacts on the global population but has also resulted in global public health emergencies due to its high infectivity and fatality rates. Although MPXV received far less attention compared to the SARS-CoV-2 pandemic due to the more severe repercussions of the latter, the occurrence of both sanitary emergencies within a relatively short time span has attracted the attention of researchers. Moreover, it is impossible to predict precisely which fatal diseases will affect the global society in the near future [6].

Given that epidemics can cause serious or permanent disorders and can rapidly spread due to their high contagiousness, it is critical to diagnose and treat viral diseases in advance. To prevent and treat the spread of epidemic diseases in their early stage, an accurate and rapid diagnosis of epidemic diseases is required. For this reason, several types of biosensors have been developed for virus detection [7,8,9]. Among these biosensors, viral nucleic acid-targeting biosensors have been developed to sensitively detect or monitor RNA/DNA viruses. The targeting of viral nucleic acids allows for the accurate diagnosis of viral diseases compared with the targeting of membrane proteins and spike proteins for the detection of RNA/DNA viruses [10]. Additionally, viral nucleic acids are attractive targets for the detection of mutations in RNA/DNA viruses, which cannot be easily achieved by using other targets [11].

Numerous biosensors have been developed to detect the nucleic acids of epidemic-causing viruses and can be acquired in the form of commercial kits. However, conventional biosensors that target viral nucleic acids have several drawbacks: (i) low selectivity and sensitivity for the accurate detection of target viral nucleic acids; (ii) limitations in the detection of mutated forms of viruses; (iii) highly time- and cost-consuming for diagnosis; and (iv) limitations on in situ monitoring for accurate detection of viruses [12,13]. Several approaches have thus been utilized to enhance the sensing performance of biosensors, but there are still many drawbacks that must be addressed [14].

The introduction of nanotechnology into the biosensing field has emerged as a promising strategy to overcome the current limitations of conventional biosensors. Nanotechnology has been applied in a broad range of scientific and medical fields due to the unique quantum mechanical properties of certain nanomaterials. Nanotechnology-assisted techniques are highly novel methods to produce nano-sized environments and their application involves the use of unique strategies. Moreover, nanomaterials have been used as significant components for several scientific and engineering research fields such as the biochemical, medical, energy, and electrical fields [15,16,17]. Thus, nanotechnology-assisted techniques and nanomaterials could greatly contribute to the development of novel viral nucleic acid biosensors. Due to their outstanding electrical, optical, and structural/systemic properties, several viral nucleic acid biosensors have been developed using nanotechnology [10,18,19]. These nanotechnology-assisted biosensors allow for the highly sensitive and selective detection of target viral nucleic acids. Moreover, given that viral mutations can be detected using nanotechnology-assisted biosensors, recent studies have developed biosensors for the early and accurate diagnosis of epidemic diseases such as SARS-CoV-2 targeting biosensors [20].

**Figure 1:**
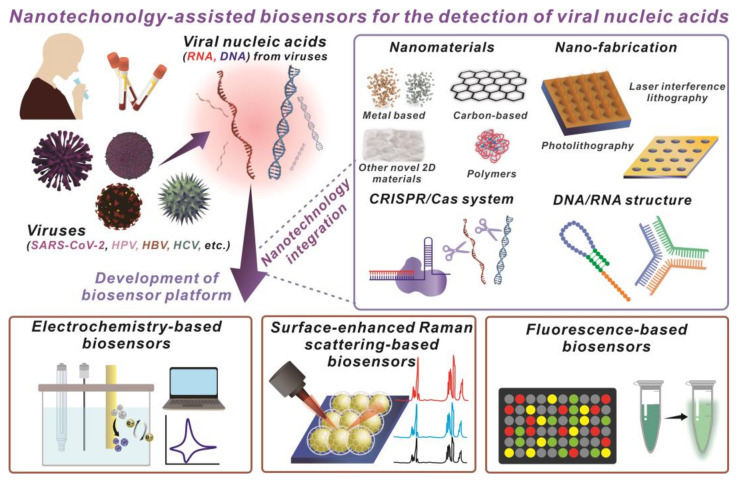
Schematic diagram of nanotechnology-assisted biosensors for the detection of viral nucleic acids.

In this review, we introduce interdisciplinary knowledge of nanotechnology-assisted viral nucleic acid biosensors (Figure 1). First, representative nanomaterials, such as metal- and carbon-based nanomaterials, and other useful materials for the fabrication of biosensors will be discussed. Each description of the nano-components for biosensors includes a summary of the advantages and disadvantages of nanomaterials to serve as a basis for the selection of appropriate materials for each intended purpose. Next, nanotechnology-assisted techniques, such as nano-fabrication techniques and nano-biotechnology, for the design and fabrication of biosensors will be discussed. Afterward, we describe nanotechnology-assisted biosensors by dividing the sections below based on different biosensing techniques such as electrochemistry, surface-enhanced Raman scattering, and fluorescence. In each of these sections, specific information on several reported viral nucleic acid biosensors will be provided in an easily understandable and applicable manner. We expect that this review will provide a comprehensive summary of interdisciplinary nano-biotechnological approaches for the effective development of novel viral nucleic acid biosensors, as well as innovative techniques for viral nucleic acid detection.

## 2. Nanotechnology Applied for Biosensors

Due to its innovative potential in a wide range of applications, nanotechnology, including nanomaterials and nanotechnology-assisted techniques, is widely considered a crucial technology in a variety of research fields. Particularly, the unique quantum mechanical properties of nanomaterials allow for the development of novel viral nucleic acid biosensors with sensitive and precise biosensing capabilities. In this section, we will focus on the numerous nanotechnologies that are currently available for the fabrication of biosensors, including nanomaterials, nanolithography, and nano-biotechnology.

### 2.1. Nanomaterials as Components of Biosensors

Nanomaterials have received considerable attention across a wide variety of scientific fields, particularly in biology-integrated areas, due to their unique characteristics, such as high conductivity and biocompatible cellular absorption. Extending the active surface area and developing novel characteristics not present in the bulk state are two of the most commonly recognized benefits of nanomaterials [21]. Due to these characteristics, nanomaterials have been applied in various approaches for the fabrication of biosensors. Several nanomaterials with highly applicable properties for the fabrication of biosensors will be introduced in this section.

#### 2.1.1. Metal-Based Nanomaterials

The use of metal-based nanomaterials in biosensing is closely linked to the surface modifications that occur after binding the biomolecular analyte and their quantum mechanical properties such as the acceleration of electrons and strong electromagnetic fields [22,23,24]. Among the various metal-based nanomaterials characterized thus far, transition metal oxides such as nickel oxide (NiO), cobalt oxide (Co_3_O_4_), and manganese oxide (MnO_2_) have been widely employed as biosensor components due to their quick and reversible Faradic redox reactions at the interface between the electrode and electrolyte [25]. For example, a bioelectrode composed of NiO and other 2D materials was used for the detection of the influenza virus [26]. In another study, Co_3_O_4_ was found to be promising for the electrochemical detection of target molecules due to its strong electrocatalytic activity, outstanding stability, and simple structural design [27]. One research group developed a Co_3_O_4_-based biosensor for the detection of miRNA-141 using an electrochemiluminescence detection technique [28]. Similar to other metal oxides, several manganese oxides are commonly employed as electrode materials in electrochemical sensing due to their catalytic properties, durability, low price, and biocompatibility [29]. MnO_2_ is one of the most prevalent manganese oxides, and it has several uses in energy storage, biosensing, and memory systems [30]. For instance, a MnO_2_ bi-electrode system was developed for the highly sensitive detection of pathogen nucleic acid [29].

**Figure 2:**
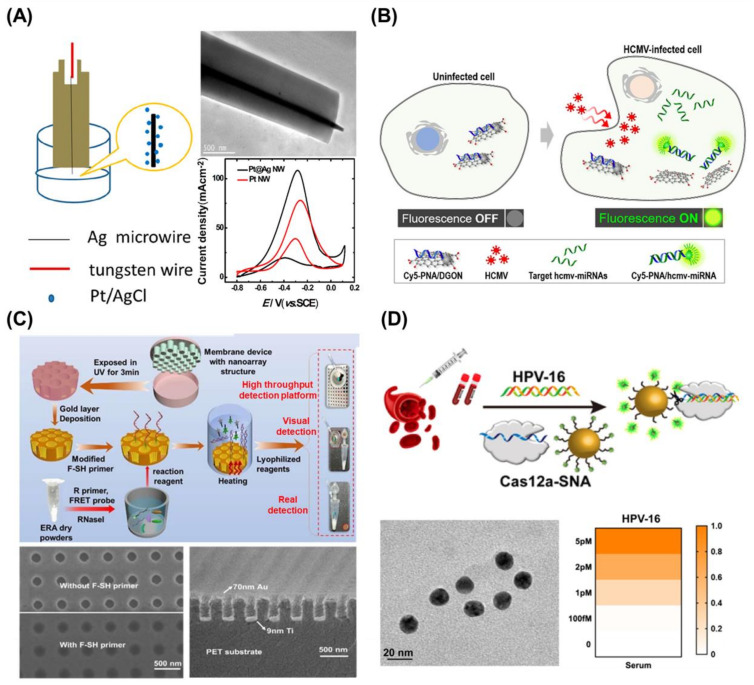
(**A**) Schematic diagram of a process for fabrication of single Pt@Ag nanowire, transmission electron microscope (TEM) image of fabricated Pt@Ag nanowire, and increase of current density of Pt nanowire after Ag modification. Reprinted with permission from ref. [35]. Copyright © 2019, American Chemical Society. (**B**) Schematic illustration of the HCMV diagnostic using dextran-coated GO nanocolloid (DGON) to detect the HCMV-encoded miRNA in the HCMV-infected living cell. Reprinted with permission from Ref. [48]. Copyright © 2021, American Chemical Society. (**C**) Schematic illustration of nanoplasmonic-enhanced isothermal amplification for SARS-CoV-2 detection and scanning electron microscope images of fabricated mold using LIL technique. Reprinted with permission from Ref. [66]. Copyright © 2022 Elsevier B.V. (**D**) Scheme of HPV-16 detection in serum samples, TEM image of AuNPs modified with reporter strands, and analysis for a heat map of different concentrations of HPV-16. Reprinted with permission from Ref. [71]. Copyright © 2021, American Chemical Society.

Since the introduction of nanomaterials in biosensor development, noble metal nanomaterials such as gold (Au), silver (Ag), and platinum (Pt) have been widely employed in the development of biosensors due to their quantum mechanical properties, relatively small size, biocompatibility, and ease of modification [31,32]. Particularly, noble metal nanomaterials can be incorporated into biosensor platforms due to their exceptional electrochemical properties, which improves both the sensitivity and selectivity of the biosensors [33]. For example, an electrochemical DNA biosensor was developed using different sizes of Au nanoparticles to take advantage of the superior electron transfer recovery ability of Au nanoparticles [34]. Similar to Au nanomaterials, Ag and Pt have also been used for the development of electrochemical biosensors. For example, a Pt/Ag nanowire electrode was fabricated to develop an electrochemical biosensor [35] (Figure 2A). Here, in the presence of Pt nanoparticles on the surface of Ag nanowires, the conductivity of the electrode increased, and the electrode showed electrocatalytic activity for the electro-oxidation of methanol. Moreover, the properties of nanowires, including their length, radius, and the ratio of noble metals, can be easily modified, thus affecting the electrical properties of the electrodes to achieve high sensitivity and selectivity.

Furthermore, noble metal nanomaterials can be applied to the development of localized surface plasmon resonance (LSPR) and surface-enhanced Raman scattering (SERS) biosensors. LSPR and SERS are the most sensitive optical biosensors for the detection of a wide variety of analytes, including ions and biomolecules. Metal-based nanomaterials are uniquely well-suited for the development of ultrasensitive plasmon-enhanced biosensors due to their dramatically amplified electric field at their sharp corners/edges under a broad range of excitation wavelengths [36]. Because of their distinctive plasmonic capabilities, noble metal nanomaterials have attracted significant attention, as they can be used to modulate the optical signal of molecules placed in their vicinity. For example, Au nanorods have been used for LSPR biosensors due to their unique optical properties such as their greater near-infrared (NIR)/Vis absorption compared to Au nanoparticles [37]. Another research group applied Ag nanopillars to detect the DNA of a target virus (avian influenza A, H9N2) [38]. Given that Ag nanopillars can be easily fabricated using a porous aluminum oxide template and tuned by changing conditions of fabrication, the detection of target virus DNA was accurately detected using the optimized effective SERS platform composed of Ag nanopillars. In summary, various metal-based nanomaterials including metal oxides and noble metals have been used for the fabrication of electrochemical and optical biosensors due to their unique properties.

#### 2.1.2. Carbon-Based Nanomaterials

Carbon-based nanomaterials have attracted great interest due to their remarkable electrical and optical properties, which makes them uniquely well-suited for biosensor development [39,40]. Since the discovery of carbon quantum dots (CQDs), several studies have been conducted to characterize their optical properties, as the photoluminescence of different types of CQDs ranges from ultraviolet to visible light and can even reach the infrared region [41]. By taking advantage of these unique optical properties, one research group fabricated a hepatitis C virus (HCV) RNA sensor using graphene quantum dots [42]. In this study, the graphene quantum dots were coated with Ag nanoclusters. Furthermore, hairpin DNA-modified magnetic nanoparticles were synthesized as separation materials and modified with glucose oxidase. In the presence of target RNA, two hairpin DNAs were hybridized, after which hydrogen peroxide was produced during glucose catalysis mediated by glucose oxidase. The resulting hydrogen peroxide changed the color of the Ag nanocluster-modified graphene quantum dot from yellow to transparent because the modified Ag nanoclusters were turned into Ag ions and superoxide. The reaction between graphene quantum dots and superoxide quenched the fluorescence through an electron charging-discharging mechanism for target detection.

Due to the electrostatic interactions between the functional groups of negatively charged single-stranded DNA molecules and the carbon nanotube (CNT) surface, electrochemical devices using CNTs for nucleic acid biosensing have grown in popularity [43]. Moreover, another study reported a CNT-based biosensor with a high surface-to-volume ratio and an electrical conductance that was particularly sensitive to the presence of surface species, which allowed it to detect single molecules [44]. For instance, a recent study described the development of a multi-walled CNT-based impedimetric biosensor for sensitive quantification of SARS-CoV-2 nucleic acids [45]. The developed biosensor allowed for the sensitive quantification of the target DNA without any amplification due to the capturing properties and conductivity of CNTs. Graphene is another material that has attracted the attention of researchers due to its extraordinary electrical, thermal, and mechanical properties, in addition to other characteristics [46]. Graphene has previously shown promise in electrochemical biosensing and wearable electronics, which could be used for the diagnosis and in situ monitoring of infectious diseases such as COVID-19. Moreover, graphene oxide (GO) has been used to develop biosensors using diverse signal transduction strategies such as optical, electrochemical, and mass spectrometric detection due to its graphene-like physicochemical features [47]. Particularly, graphene has been widely utilized for the development of fluorescence resonance energy transfer (FRET) biosensors due to its unique role as a fluorescence quencher. Moreover, GO can interact with diverse biomolecules through pi-pi interactions and hydrogen bonding, thus binding to single-stranded DNA with strong affinity rather than double-stranded DNA. Accordingly, a GO-based viral miRNA biosensor was developed by taking advantage of the unique properties of graphene materials. In this study, human cytomegalovirus (HCMV)-infected cells were treated with newly fabricated dextran-coated GO nano colloids with fluorescence peptide nucleic acids for the detection of HCMV-miRNA [48]. In the presence of the target HCMV-miRNA, the target miRNA was hybridized with the peptide nucleic acids and the separation of fluorescence peptide nucleic acids from GO, which has a quenching effect, significantly increased the fluorescence signal (Figure 2B).

As discussed in this section, carbon-based nanomaterials have been widely employed as components for the development of many types of biosensors due to their exceptional features, including strong electrical conductivity, binding affinity for nucleic acids, and involvement in quenching effects [49]. Given that the sizes and structures of carbon-based nanomaterials can be easily modified and customized, these materials offer a promising basis for the future development of biosensor platforms using a variety of techniques.

#### 2.1.3. Other Nanomaterials

In addition to the nanomaterials described above, other types of nanomaterials have been used to develop biosensors due to their specific characteristics. In contrast to conventional fluorescent probes, an upconversion nanoparticle (UCNP) is a suitable energy donor in biosensors due to its non-autofluorescence, good stability, great light penetration depth, and extended lifespan. For example, a recent study developed a UCNP-based virus-specific nucleic acid biosensor to detect nucleic acids [50]. Moreover, a NaYF_4_:Yb, Er@NaYF_4_ core/shell UCNP was synthesized and immobilized onto an Au nanoparticle-modified quartz glass plate. In the absence of the target DNA, luminescence resonance energy transfer occurred between the UCNP and the Au nanoparticles, thereby quenching the upconversion luminescence. Conversely, in the presence of the target DNA, since the target DNA hybridized with hairpin-structured DNA on the UCNP, luminescence was recovered due to the increase of distance between the UCNP and the Au nanoparticle through the formation of double-stranded DNA. Additionally, graphene-like 2D materials such as transition metal dichalcogenides (TMD) and transition metal carbides, nitrides, or carbonitrides (Mxene) [51] have attracted the attention of researchers in the biosensor field because (i) their electronic signals are highly enhanced through ideal electron transfer carriers and (ii) their unique physical properties such as their mechanical strength, light transmission from strong covalent bonds, and the thickness of the atomic layers [52]. Due to its metallic nature, layered 2D molybdenum disulfide (MoS_2_) is compatible with electrochemical systems and has been employed as an electrode material in a number of sensing applications [53]. Based on these advantages, MoS_2_ was utilized to fabricate a biosensor for the detection of human papillomavirus (HPV) [54]. After modifying the aptamer on the electrodes, the fabricated biosensor could sensitively detect HPV-specific nucleic acids in human serum and saliva samples due to the electronic properties and the large surface area of 2D materials.

Furthermore, MXene, a novel 2D material, has recently garnered increasing interest in the biosensing field due to its layered structure, ease of functionalization, and good metal conductivity [55]. Utilizing the advantages of Mxene, a Mxene-based biosensor for SARS-CoV-2 detection was fabricated [56]. In this study, the Mxene demonstrated a significant inherent photoelectric conversion impact and enabled the effective modification of numerous functional groups on its surface. Therefore, the Mxene was modified on Au nanoparticles with three-stranded complex DNAs. In the presence of target DNA (*RdRp* gene from SARS-CoV-2), fuel DNA, and walking DNA, one DNA strand in a three-stranded DNA structure was hybridized with the fuel DNA. The hybridized DNA complex activated the CRISPR/Cas12a system, which in turn cleaved the single-stranded DNA with ferrocene, thereby quenching the signals on the electrode. Eventually, the electrodes exhibited electrochemiluminescence signals for the detection of the *RdRp* gene. In another study, boron nitride quantum dots (BNQDs) were applied for the fabrication of a biosensor due to its unique properties such as a high quantum field and stability [57]. Additionally, conducting polymers, such as polyaniline (PANI), polypyrrole (PPy), and poly(3,4-ethylenedioxythiophene) (PEDOT), can be suitable candidates for components of biosensors due to their conductivity, biocompatibility, and tunable mechanical properties [58]. For example, one research group developed a PPy-based biosensor for the detection of miRNA-21. In this study, Ppy was combined with graphene to enhance the electrochemical properties of the biosensors. Through conjugation with Au nanoparticles and the Ppy/graphene complex, the electrochemical signal from methylene blue (MB) increased on the fabricated biosensor. The fabricated biosensor exhibited highly sensitive detection of target RNA because the MB could intercalate more efficiently with double-stranded DNA, thus promoting the hybridization with target RNA and probe RNA on electrodes.

Various nanomaterials have attracted widespread attention and have been utilized by researchers in the biosensor field due to their unique properties. However, there are still a large number of potential applications of nanomaterials for biosensor development, which not only include the above-described nanomaterials but also emerging nanomaterials such as liposome nanoparticles, dendrimers, and others [59]. With the incorporation of novel nanomaterials, future biosensors could overcome the current limitations on the detection of target nucleic acids such as their narrow detection range, the need for complex systems for selective sensing, and the detection of mutated nucleic acids.

### 2.2. Nano-Fabrication Techniques and Nano-Biotechnology for Biosensors

Various nanotechnology-assisted methodologies have been developed as a result of the advancement of nanotechnology to enable the development of biosensors. These novel methodologies have increased the performance of biosensors by structural merits and facilitated the creation of complex combinations of sensing platforms employing biomaterials, thus expanding the sensing range of biosensors, and enabling the detection of numerous targets. This section will cover the nano-fabrication techniques and nano-biotechnologies utilized in the development of biosensors.

#### 2.2.1. Nano-Fabrication Techniques

Lithographic techniques are commonly employed for the creation of biosensors because they can produce complex micro/nanoscale structures with high-resolution topography, in addition to enabling the localized deposition of soft molecules while preserving their bioactivity [60]. Additionally, nanostructures composed of functional nanomaterials have several advantages associated with their electrical and optical properties due to their higher surface area compared with flat surfaces, as well as the tunability of their optical properties by modifying their size and shape.

Photolithography is the most widely used technique for micro- and nano-fabrication [61]. For example, a microfluidic device with a droplet generator and a picoinjector was fabricated utilizing photolithography [62]. By applying the photolithography method, the microfluidic chip was successfully developed, and the compartmentation/reaction of target HPV DNA from the sample was achieved using the microfluidic chip. Then, only droplets containing the target DNA underwent amplification and exhibited fluorescence signals. Even though the microfluidic chips had a sophisticated structure and design for accurate biosensing, their implementation still had several limitations such as the requirement of specialized instruments for their fabrication, challenges in nano-size fabrication, and high costs.

Electron beam lithography (EBL) is another lithography technique that has been employed to obtain customized nanopatterns with nanometer-scale resolutions on a flat surface covered with electron beam-sensitive resist materials by focusing an electron beam [63]. EBL has also been applied to develop biosensors because it allows for ultra-small-sized nanopattern generation. A meticulously designed field effect transistor (FET) biosensor for the hepatitis B virus (HBV) was fabricated using EBL [64]. Titanium/Au (20/60 nm) electrodes with 300 nm of spacing were successfully fabricated for the delicate source and drain electrodes of the FET biosensor due to the high resolution of EBL for nano-fabrication. Afterward, the biosensor’s performance was assessed by analyzing PCR-verified samples. The fabricated FET biosensor could effectively discriminate between healthy and normal samples, thus demonstrating its great accuracy and specificity. However, the widespread adoption of the EBL technique continues to be limited due to its high costs and long fabrication times.

Additionally, laser interference lithography (LIL) is a nanolithography method that uses laser interference. Due to its size-tunability, manufacturing speed, and uniformity over a large interfacial area, this technique has been widely adopted in several fields, including biosensors [65]. For instance, a recent study described the construction of an ultra-sensitive nanoplasmonic sensor for the detection of SARS-CoV-2 nucleic acids using the LIL technique [66]. Using this technique, 200 nm-sized diameter nanoplasmonic chip arrays were uniformly fabricated (Figure 2C). By applying the FRET biosensing technique, the fabricated biosensor demonstrated highly sensitive, rapid, high efficiency, and accurate screening capacity for SARS-CoV-2 viral detection. Nevertheless, depending on the instrument settings (e.g., the wavelength of lasers), the LIL technique may not be feasibly applied to fabricate complex nanostructures.

#### 2.2.2. CRISPR/Cas System-Based Nanotechnology

Clustered regularly interspaced short palindromic repeats and Cascade proteins (CRISPR/Cas)-based biosensing, which is known for its capacity to recognize and cleave specific DNA or RNA sequences, has emerged as a novel diagnostic tool for nucleic acid detection, in addition to its well-known gene editing applications. Among the current complex and evolving CRISPR/Cas systems, the Class 2 family, which is characterized by a single Cas effector and includes Cas9, Cas12, and Cas13, appears to have greater potential for nucleic acid detection, as the participation of a single Cas protein facilitates the development of reliable CRISPR/Cas-based biosensors [67,68]. In addition to the sensitive recognition and cleavage of target DNAs or RNAs, the unique collateral activity of Cas12a and Cas13 systems enables the construction of highly sensitive nucleic acid detection methods. The effective recognition of the target nucleic acid by the Cas12a and Cas13 systems produces a trans-cleavage catalytic reaction, and therefore, this approach is uniquely well suited for the detection of signal-amplified nucleic acids [69,70]. Cas12a and Cas13-based biosensors convert the trans-cleavage activity into observable optical or electrochemical signals. Based on this principle, a CRISPR/Cas12a-based fluorescence biosensor for the detection of HPV-16 was fabricated by coupling the CRISPR/Cas12a system with a spherical nucleic acid reporter [71] (Figure 2D). In the presence of the target DNA, the fluorescence dye-modified DNA on an Au nanoparticle was trans-cleaved by the activated Cas12a complex. In turn, HPV-16 was successfully detected by quantifying the resulting fluorescence signals. The effective application of the CRISPR/Cas system for nucleic acid detection in real clinical samples also demonstrated its promising potential for future biology research and medical diagnosis. With the creation of CRISPR/Cas-based diagnostic platforms, the SHERLOCK (specific high-sensitivity enzymatic reporter unlocking), miSHERLOCK, streamlined highlighting of infections to navigate epidemics (SHINE), and Cas13-based, rugged, equitable, scalable testing (CREST) platforms have been developed, and the versatility of CRISPR/Cas-based biosensing technology for SARS-CoV-2 detection has been confirmed [72]. Although CRISPR/Cas-based biosensors have been confirmed to detect single molecule targets with high selectivity and sensitivity, the target nucleic acids must be amplified to enhance the sensing sensitivity.

#### 2.2.3. DNA/RNA Structure-Based Nanotechnology

DNA nanotechnology employs the base-pairing language to create nanostructures and manipulate matter at the nanoscale level, and this technique has been extensively used in various fields including nanomachines, gene editing, molecular computing, targeted medication delivery, and biosensing [73,74]. The programmable construction of DNA nanostructures has recently emerged as an adaptive instrument for improving the performance of single-molecule sensing and imaging. The binding of nucleic acid structures to target analytes induces structural changes in the nucleic acid structures, which are employed as signal readouts through the hybridization of the nucleic acids [75]. For example, an electroluminescence biosensor to detect tobacco mosaic virus RNA (tRNA) was developed using hybridization between target tRNA and probe hairpin DNA [76]. In the presence of the target tRNA, the probe hairpin DNA modified on the electrodes changes its form through hybridization. Consequently, the functional group at the end of the probe hairpin DNA was kept away from the electrode surface. Afterward, polymer groups were generated from the functional group and luminol was attached to the generated polymer groups, resulting in the successful detection of the target tRNA. In addition to the hairpin-shaped DNA, numerous other types of DNA structures have been implemented in biosensors for the detection of target nucleic acids with functional expansion. For instance, custom-designed Y-shaped DNA dual probes were utilized for the detection of SARS-CoV-2 nucleic acids [77]. Due to the synergistic interaction of the two probe sites at the Y-shape DNA dual probe targeting the two different regions of SARS-CoV-2, the fabricated biosensors exhibited a greater recognition ratio toward SARS-CoV-2 nucleic acids. Moreover, nucleic acid extraction, polymerase chain reaction (PCR), or other reaction-based amplification were not required to accomplish high sensitivity and rapid detection using the fabricated biosensor platform. Similarly, G-quadruplex DNA has attracted considerable attention because it can be employed for both signal transduction through metal binding and sensitive target recognition [78].

In summary, numerous biosensors have been fabricated in recent years by applying the unique structure of nucleic acids. The complex structural modifications of nucleic acids may improve the sensitivity of biosensors, but they may also increase the total size and complexity of the sensors, thus limiting their fabrication and commercialization potential.

#### 2.2.4. Target Nucleic Acids Amplification Techniques

To precisely detect the target viral nucleic acids, nucleic acid amplification techniques (NAATs) such as isothermal amplification and rolling circle amplification (RCA) are powerful tools that can improve the sensitivity of biosensors [79]. For example, a human immunodeficiency virus (HIV)/HCV biosensor was developed utilizing Exo III-assisted target recycling amplification (ERA) and RCA techniques [80]. In the initial step of amplification, the ERA amplification products were employed as a primer to initiate the subsequent RCA process, thereby optimizing the operation. Additionally, the RCA products provided templating sites for the in situ development of Ag nanoclusters, which produced a fluorescent signal for label-free detection. Even though NAATs can enhance biosensor sensitivity, there are still some drawbacks that complicate the diagnostic procedure, including the RNA extraction requirements, off-target amplification, and amplification times.

## 3. Electrochemistry-Based Biosensors for the Detection of Viral Nucleic Acids

**Figure 3:**
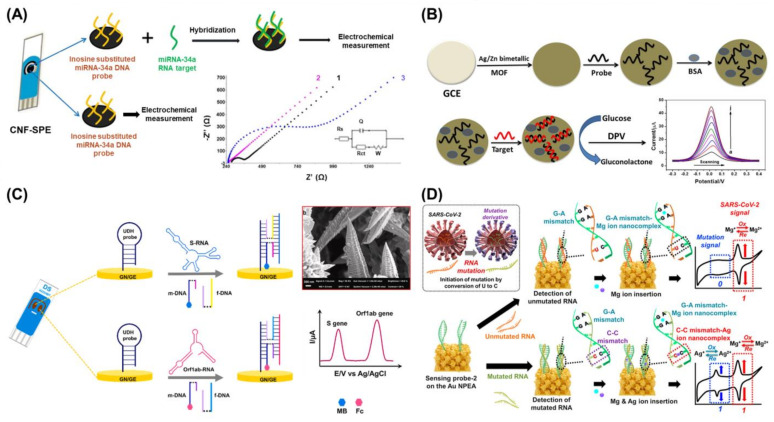
(**A**) Schematic diagram of indicator-free electrochemical biosensor for miRNA-34a detection, and Nyquist plot results of bare SPE (1), CNF-SPE (2), and after detection of miRNA-34a (3). Reprinted with permission from Ref. [84]. Copyright © 2015 Elsevier B.V. (**B**) Schematic diagram of fabrication process of the bimetallic Ag/Zn-MOF-based electro-chemical HCV RNA biosensor, and amplification-assisted HCV RNA detection using DPV technique. Reprinted with permission from Ref. [88]. Copyright © 2021 Elsevier B.V. (**C**) Schematic illustration of an electrochemical SARS-CoV-2 biosensor on the GN/SPGE using 4-WJ structure of nucleic acids and scanning electron microscopy (SEM) images of fabricated GN. Reprinted with permission from Ref. [89]. Copyright © 2021 Elsevier B.V. (**D**) Scheme of determination of single point mutation occurrence of SARS-CoV-2 viral RNA using MNM nanocomplexes on the NPEA. Reprinted with permission from Ref. [20]. Copyright © 2021 Elsevier B.V.

Electrochemistry is one of the most widely used techniques for biosensor development [81,82]. In general, electrochemical biosensors are sensitive to the electrochemical reactions between targets and probes or the changes in the electrochemical signals from redox molecules that appear or disappear after target detection [83]. Particularly, depending on the type of target to be measured or the measurement environment, various electrochemical technologies can be selected and implemented for the development of appropriate electrochemical biosensors. Accordingly, various electrochemical techniques such as cyclic voltammetry (CV), amperometry, electrochemical impedance spectroscopy (EIS), and differential pulse voltammetry (DPV) have been appropriately utilized to develop various types of electrochemical biosensors based on specific targets and sensing environments. In the case of biosensors targeting nucleic acids, since nucleic acids have no conductivity or electrochemical characteristics, EIS and impedance techniques have been used to develop biosensors by measuring increases in the electrochemical resistance of the non-conductive layer formed by capturing the target nucleic acid. Based on this biosensing process, an electrochemical indicator-free voltammetric biosensor was developed using carbon nanofibers (CNFs) for the detection of miRNA-34a [84] (Figure 3A). In this study, the authors developed a CNF-enriched disposable screen-printed electrode (CNF-SPE), which was then applied as an electroconductive electrode with outstanding mechanical and thermal properties for the modification of DNA biosensing probes and miRNA detection. As a DNA probe, the amine-modified complementary DNA sequence of miRNA-34a was immobilized on the surface of the CNFs via the covalent bonding between the carboxyl groups of the CNF and the amine group of the DNA probe. Then, through the detection of miRNA-34a by a DNA probe, the increased resistance caused by the formation of a double-stranded nucleic acid structure was successfully monitored by EIS and DPV without the introduction of any redox indicators following a simple detection approach. As shown in Figure 3A, the charge transfer resistance (R_ct_) value increased after miRNA-34a detection.

As mentioned in Section 2, the incorporation of nanotechnologies or nanomaterials can significantly increase the biosensing abilities of biosensors. One of the most important factors when developing an electrochemical biosensor is to improve the conductivity of the electrode to facilitate electron transfer reactions between the probe and target nucleic acids, thus enabling highly sensitive target detection [85]. By taking advantage of the unique conductive properties and large surface areas of nanomaterials or nanopatterned arrays on electrode surfaces, numerous nanomaterials and nanotechnologies have been used to develop superior electrochemical viral nucleic acid biosensors [86,87]. For instance, a silver/zinc bimetallic metal-organic framework (Ag/Zn-MOF) was fabricated and used as an electrode to develop a label-free electrochemical HCV biosensor [88]. The resulting bimetallic Ag/Zn-MOF, which was cast on a glassy carbon electrode (GCE) with an HCV-capturing DNA probe, provided a large surface area, superior catalytic activity, and a protection layer for the DNA probe. Furthermore, due to the introduction of the Ag/Zn bimetallic structure, this MOF could overcome the limitations of conventional MOFs with low conductivity. Moreover, electrochemical signal amplification was achieved via the catalytic reaction of the bimetallic Ag/Zn-MOF in the presence of glucose for the highly sensitive detection of HCV RNA with the developed biosensor (Figure 3B). In another study, an amplification-free electrochemical SARS-CoV-2 biosensor was developed on a 3D Au nanoneedle structured electrode [89]. In this study, a four-way junction (4-WJ) structure of nucleic acids was designed to rapidly detect both SARS-CoV-2 spike (S) and open reading frame (*Orf1ab*) genes. In this 4-WJ assisted biosensing system, a universal DNA hairpin (UDH) probe and another two types of DNA adaptors were introduced for capturing *S* or *Orf1ab* genes, respectively, after which the 4-WJ structure was formed. Since one of the two DNA adaptors was modified with MB or ferrocene (Fc), the detection of the S and *Orf1ab* genes was easily confirmed through the measurement of redox signals from MB or Fc (Figure 3C). Furthermore, to improve the electrochemical signals from redox molecules in the 4-WJ structure after the detection of the SARS-CoV-2-specific genes, a screen-printed Au nanoneedle electrode (GN/SPGE) was developed using an electrodeposition technique. By introducing novel nucleic acid structure-based biosensing processes and nanotechnology-based electrodes, the developed biosensor could sensitively detect both the *S* and *Orf1ab* genes [limit of detection (LoD): 5.0 and 6.8 ag/μL, respectively].

Additionally, previous studies have developed lab-on-a-chip electrochemical biosensors for viral nucleic acids by implementing functional or conductive nanostructures or an electrode system on a chip using nanotechnologies such as photolithography and printing technology [90,91,92]. In one of these studies, a microfluidic- and photolithography-assisted lab-on-a-chip system was developed for the multiplexed electrochemical detection of SARS-CoV-2 RNA and anti-SARS-CoV-2 antibodies [92]. Moreover, a nanotechnology- and biotechnology-assisted electrochemical biosensor for the facile detection of SARS-CoV-2 gene mutations was recently reported [20]. Here, the authors employed mismatched nucleic acid–metal ion (MNM) nanocomplexes as a biosensing mechanism to forego complex pre-treatment processes such as PCR and additional redox molecule tagging steps. Generally, under simple lab-scale conditions for biosensor fabrication, the attachment of redox or fluorescent chemicals to nucleic acids is commonly outsourced, which increases fabrication times and costs. To avoid these additional processes, a bio-nanocomplex integrating nanotechnology and biomaterials was utilized as a key component. When there is a mismatched nucleic acid sequence in double-stranded sequences, specific metal ions can intercalate in the mismatched sequence to stabilize the overall structure. Inspired by this natural phenomenon, Ag and Mg ions, and cytosine-cytosine (C-C) and guanine-adenine (G-A) mismatches were used to form the MNM nanocomplexes (C-Ag-C and G-Mg-A) after viral RNA detection using custom designed DNA probes. Furthermore, unlike conventionally used redox molecules such as MB and Fc, the intercalated metal ions exhibit redox characteristics. Therefore, single-point mutations in SARS-CoV-2 viral RNA were detected using two MNM nanocomplexes (Figure 3D). Moreover, to improve the sensitivity of this biosensor by providing a conductive layer with a high surface-to-volume ratio, a nanoporous electrode array (NPEA) was developed using the LIL technique and electrochemical deposition. The developed biosensor could not only be used as a basis for the development of easy-to-use biosensors for point-of-care testing but also for the facile detection of viral mutations.

The development of electrochemical viral nucleic acid biosensors using nanomaterials and CRISPR/Cas has also attracted increasing attention in recent years [93,94]. Particularly, these biosensors are ultra-sensitive because the CRISPR/Cas system can recognize RNA or DNA with extremely high specificity. For example, using the specific RNA sequence recognition ability and trans-cleavage property of CRISPR/Cas13a for SARS-CoV-2 RNA detection, an electrochemical SARS-CoV-2 RNA biosensor was developed on a nanomaterial-decorated electrode composed of an Au nanoflower, MoS_2_ nanosheet, and Au nanoparticles [94]. In this study, a redox molecule-modified RNA probe was immobilized on a nanomaterial-decorated electrode as a probe molecule. In the presence of SARS-CoV-2 RNA, the activated CRISPR/Cas13a cleaved the RNA probe on the electrode, thus enabling the precise detection of SARS-CoV-2 RNA by sensing the disappearance of redox signals. By coupling the sensitive detection properties of CRISPR/Cas13a and electrochemically active electrodes, this sensor achieved ultrasensitive detection with LoD values below the fg/mL level.

Although flexible or wearable electrochemical biosensors have garnered increasing interest in recent years, their widespread adoption has been limited by the inherent drawbacks of electrochemical biosensors, including the need for a two-electrode or three-electrode system and an electrolyte container. Nevertheless, if only a few drawbacks can be addressed, nanotechnology-assisted electrochemical biosensors could become a promising basis for the development of viral nucleic acid biosensors that can be practically applied [95].

## 4. Surface-Enhanced Raman Scattering-Based Biosensors for the Detection of Viral Nucleic Acids

Raman spectroscopy is a potential analytical method that currently offers a chemical fingerprint that may be used for the identification of molecules [96]. The Raman spectroscopy technique makes use of light that has been inelastically dispersed and enables the determination of the vibrational states (phonons) of molecules. Given that the spectrum of scattered photons is unique for each molecule, the discovery of various types of Raman techniques contributed greatly to the biomedical field and applied research [97]. Raman spectroscopy allows for the quick and easy detection of target molecules. Moreover, in addition to being unintrusive, this technique can be used with aqueous materials, does not require sample preparation, and can be combined with other analytical methods. Due to its unintrusive nature, Raman spectroscopy can be used for in vivo analysis and diagnosis. Additionally, the Raman spectrum can provide information about the structure, conformation, and interaction of biomolecules [98]. Surface-enhanced Raman scattering (SERS) is a spectroscopic detection technique that has recently attracted great interest in the field of biomedicine [99]. SERS creates a highly intense electromagnetic field at the nanoscale that can significantly increase the Raman scattering intensity by making use of the unique optical characteristics of metallic nanostructures [100]. For example, SERS is superior to traditional Raman scattering because it can enhance the Raman signal by up to 14-fold, thus allowing for ultrasensitive detection of target molecules [101]. This technique has thus been implemented in a large number of biosensors for the detection of viral nucleic acids.

**Figure 4:**
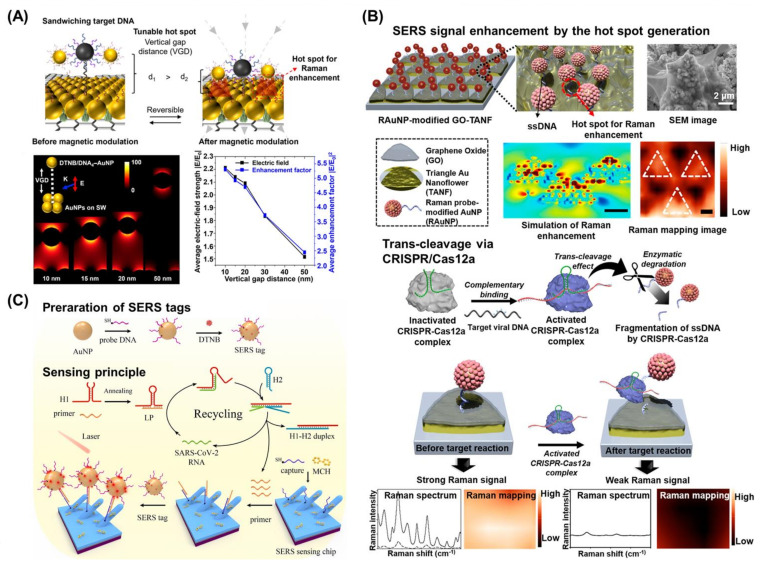
(**A**) Schematic diagram of Raman enhancement using magnetic modulation. Finite-Difference Time-Domain (FDTD) simulation results for change of vertical gap distance. From the calculation of electric field strength and enhancement factor, Au nanoparticle models showed ~1.5 fold and ~2.0 fold increments from 50 nm to 10 nm of vertical gap distance, respectively. Reprinted with permission from Ref. [18]. Copyright © 2022, American Chemical Society. (**B**) Fabricated platform composed of GO and periodic triangle nanoflower array with Raman probe modified Au nanoparticles enhanced the SERS signal by the generation of the hot spot, and after trans-cleavage via CRISPR/Cas12a in the presence of target nucleic acids, the Raman signal decreased since Raman probe modified Au nanoparticles were separated. Reprinted with permission from Ref. [104]. Copyright © 2021, American Chemical Society. (**C**) Schematic diagram of the preparation of SERS tags with Au nanoparticles and the catalytic hairpin assembly (CHA)-based signal recycling amplification method for SERS detection of SARS-CoV-2 RNA. Reprinted with permission from Ref. [107]. Copyright © 2022 Elsevier B.V.

To enhance the Raman scattering intensity, various types of nanomaterials and nanostructures were used to fabricate the biosensors. The LSPR of metallic nanostructures such as Au nanoparticles can enhance electromagnetic fields. At nanoscale junctions, intense LSPR fields known as “hot spots” can dramatically amplify the SERS signal [102]. For instance, a biosensor composed of reduced GO and Au nanoparticles was fabricated to detect SARS-CoV-2-specific nucleic acids [18]. In this study, the authors described an approach that allowed for the modulation of the distance between Au nanoparticles to enhance the electromagnetic field in a SERS hot spot after capturing the target viral nucleic acids, especially when using stimuli-responsive platforms. Two Au nanoparticles with two distinct DNAs that were complementary to the respective positions of the target nucleic acid were sandwiched between a target nucleic acid. In the hot spot region, the SERS signal of the Raman tags that were conjugated to either of the Au nanoparticles was amplified (Figure 4A). In the presence of the target DNA (i.e., the SARS-CoV-2 mimetic N protein sequence), the fabricated biosensor showed high selectivity and the authors observed a linear relationship between the Raman signals and the logarithmic picomolar concentration of the target DNA with a 1 fM LoD, thus confirming the high sensitivity of their proposed approach. In addition to Au nanoparticles, Ag nanoparticles such as Ag nanostars, Ag triangular nanoplates, or Ag nanocubes can also greatly improve SERS intensities because of the strong SERS signals from Ag and its unique structural properties [103]. An Ag nanoparticle-based biosensor was fabricated and SARS-CoV-2-specific nucleic acid targets (*RdRp* gene, *E* gene, *N* gene) were detected by the biosensor. Additionally, the highly stable Ag nanoparticles in the sample solution were averaged out to analyze the SERS response, which greatly increased the reproducibility of signals of the fabricated platform. Therefore, these findings demonstrated that the incorporation of nanomaterials and nanostructures enhances SERS intensity, thus enabling the sensitive detection of target nucleic acids.

The CRISPR/Cas system has also been applied to SERS-based biosensors in recent years. Due to the strong intensity of Raman probes on a SERS platform, SERS-based biosensors have been developed using the CRISPR/Cas system. When coupled with probes, this system can cleave specific nucleic acid sites, after which a decrease in SERS intensity in the presence of the target nucleic acids can be observed and measured. For example, the fabrication of an amplification-free viral DNA biosensor using a SERS analytical system with CRISPR/Cas12a guidance was reported [104] (Figure 4B). The fabricated biosensor was composed of GO and a periodic triangle nanoflower array with Raman probe-modified Au nanoparticles. In this study, the periodic triangle nanoflower array improved the Raman signal from the Au nanoparticles due to the highly concentrated surface electrons on the surface of the biosensor. In the absence of target nucleic acids (HBV DNA), the biosensor exhibited strong Raman intensity due to the enhanced SERS effect from nanomaterials and the nanostructures of the biosensors. In contrast, in the presence of target nucleic acids, CRISPR/Cas12a was activated and cleaved single-stranded DNA which connects the periodic triangle nanoflower array and Raman probe-modified Au nanoparticles. Therefore, the biosensor showed a decrease in the Raman signal due to the separation of Raman probes from the substrate. Utilizing the fabricated biosensor, sensitive detection of target HBV DNA was achieved with LoD values as low as 1 aM, a wide linear dynamic range (from 1 aM to 100 pM), short reaction time (20 min), and the possibility of multiplexed detection. Likewise, using the unique mechanism of the CRISPR/Cas system, an aggregated Au nanoparticle-based biosensor was fabricated [105]. Given that aggregated Au nanoparticles possess a stronger Raman signal than a single Au nanoparticle, the dispersed form of the Au nanoparticles, which lacked a linker single-stranded DNA cleaved by CRISPR/Cas, showed weak Raman intensity. Furthermore, using a specially designed chimeric DNA/RNA hairpin structure, a CRISPR/Cas system-based “On-Off” SERS biosensor was developed [106].

Moreover, a SERS-based biosensor powered by non-enzymatic signal amplification was developed using the hairpin structures of specially designed DNAs [107]. On an Ag nanorod arrayed SERS sensing chip, the sensing performance of the fabricated biosensor was confirmed by conducting a one-pot hybridization of the lock probe, hairpin DNAs, and SERS tags with SARS-CoV-2 RNA samples for the detection of SARS-CoV-2 RNA (Figure 4C). Using catalytic hairpin assembly (CHA)-based signal amplification, the capture of SERS tags on the SERS sensing chip was enhanced and an improved SERS signal was generated for the sensitive detection of SARS-CoV-2 RNA. The developed SERS-based biosensor for SARS-CoV-2 RNA detection showed rapid detection within 50 min with a low LoD (51.38 copies/mL) and wide linear detection range (102–106 copies/mL), and exhibited good detection uniformity, stability, and ability to accurately discriminate between SARS-CoV-2 RNA and RNA from other respiratory viruses.

Although SERS-based biosensors are extremely sensitive for viral nucleic acid detection, there are several drawbacks that must be overcome. For example, this approach requires large and complex instruments to observe the Raman signal, and therefore this approach does not enable the facile detection of viral nucleic acids. Additionally, the fabrication steps would be more complicated than those for fluorescence-based biosensors because most SERS-based biosensors are utilized as substrate-based biosensors rather than solution-based. With the miniaturization and integration of SERS technology with other novel nanotechnologies, improved SERS-based biosensors could be utilized as useful viral nucleic acid biosensors in a user-friendly and simple manner.

## 5. Fluorescence-Based Biosensors for the Detection of Viral Nucleic Acids

**Figure 5:**
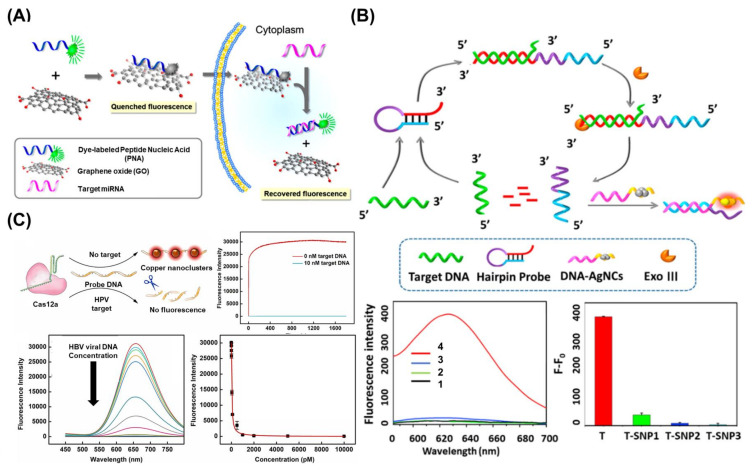
(**A**) Schematic illustration of PANGO-based fluorescent biosensor for detection of miRNAs in living cells. Reprinted with permission from Ref. [110]. Copyright © 2013, American Chemical Society. (**B**) Schematic diagram of biosensing mechanism of fluorescent biosensor composed of DNA-AgNCs, the G-rich hairpin probe and Exo III for detection of HIV-1 DNA, the fluorescence intensity analysis results of this biosensor for HIV-1 detection [(1) only DNA-AgNCs, (2) without HIV-1 DNA, (3) without Exo III, and (4) developed biosensor with HIV-1 DNA], and for distinguishing the HIV-1 DNA and nucleic acids in which some sequences were mismatched from HIV-1 DNA [T: HIV-1 DNA, T-SNP: single-base pair mismatched DNA, T-SNP2: double-base pairs mismatched DNA, and T-SNP3: triple-base pairs mismatched DNA]. Reprinted with permission from Ref. [115]. Copyright © 2021, American Chemical Society. (**C**) Schematic illustration of a “turn-off” type fluorescent biosensor composed of CRISPR/Cas12a, single-stranded DNA probe, and metal NCs for detection of HBV viral DNA, and its fluorescent signal analysis results with high sensitivity. Reprinted with permission from Ref. [122]. Copyright © 2022 Elsevier B.V.

Fluorescence is among the most widely used techniques for the development of viral nucleic acid biosensors because it can intuitively be used for disease diagnosis and confirmation [108,109]. Fluorescent biosensors generally implement a diagnostic function by confirming the appearance or disappearance of a fluorescence signal through a binding reaction between a target and a probe coupled with a fluorescence molecule [47]. Most fluorescence-based biosensing processes share the same fundamental principles. In the absence of a target, fluorescence is blocked when a quenching molecule and a fluorescence molecule are immobilized on both ends of the probe. However, in the presence of the target, fluorescence is recovered when the quenching molecule separates from the fluorescence molecule on the probe via the binding of the target and a probe. Therefore, target detection can be evaluated by measuring the fluorescence intensity. For instance, in one study, a peptide nucleic acid and nano GO (PANGO) modified with a fluorescence molecule was used as a fluorescence probe for miRNA detection [110]. In this study, in the absence of target miRNA, the fluorescence signal was quenched because the fluorescence molecule-modified peptide nucleic acid was located close to the nano GO, which plays a role as a quencher. Conversely, when the target miRNA was present, the fluorescent peptide nucleic acid was released from the nano GO through the formation of double-stranded structures, and the fluorescence signal is recovered. Based on this sensing process, a PANGO-based sensor successfully detected several miRNAs including miRNA-21, miRNA-125b, and miRNA-96 from MDA-MB-231cells (Figure 5A). Particularly, this fluorescence biosensor has huge potential in the development of CRISPR/Cas-assisted fluorescent biosensors, in which CRISPR/Cas is activated after binding with a target and can degrade the probe through site-specific cleavage [111]. Moreover, CRISPR/Cas-assisted fluorescent biosensors allow for multiplexed detection via the simultaneous introduction of various fluorescence molecules [112,113].

Similar to how nanotechnology and nanomaterials contributed to the improvement of conductivity in electrochemical biosensors, they have also been used in the development of fluorescent biosensors through the enhancement of fluorescence signals or the production of functional probes. Particularly, metal-enhanced fluorescence (MEF), in which fluorescence intensity is greatly enhanced when the distance between the metallic surface and the fluorescence molecules is a few nanometers, is widely used to improve the sensitivity of fluorescent biosensors. Furthermore, the FRET technique, which uses an energy transfer between two different fluorescence molecules, is widely used in the same context [114]. Additionally, some metal nanomaterials emit fluorescent signals and can therefore be used directly to develop biosensors without the need to introduce additional fluorescent molecules into the biosensors. In one study, the authors fabricated fluorescent and photostable Ag nanoclusters (AgNCs) to develop a fluorescent biosensor for the detection of viral nucleic acids of SARS-CoV-2, HIV, and influenza virus [115]. Moreover, through the modification of DNA on the surface of AgNCs (DNA-AgNCs), their fluorescence intensities increased drastically due to the protection provided by the DNA surrounding the AgNCs. In another study, the fluorescence intensity and emission wavelengths of AgNCs could be fine-tuned through the modification of introduced DNA structures, synthesis conditions, and the introduction of additional chemical components. Based on the prepared DNA-AgNCs, a G-rich hairpin probe and exonuclease III (Exo III) were used to demonstrate the ability of the biosensor to detect viral nucleic acids (Figure 5B). In the presence of target viral nucleic acids, the hairpin probe was opened, and the double-stranded region was degraded by Exo III. Then, the prepared DNA-AgNCs were hybridized with the remaining single-stranded fragments, resulting in strong fluorescent signals from the AgNCs. Using this biosensing mechanism, the developed biosensor successfully detected viral nucleic acids of SARS-CoV-2, HIV-1, and influenza virus. Moreover, due to its highly sensitive detection properties, the developed biosensor distinguished the target viral nucleic acid and nucleic acids in which some sequences were mismatched. Additionally, another study described the construction of a fluorescent SARS-CoV-2 biosensor composed of DNA-conjugated CdTe quantum dots and BHQ2 quencher-modified DNA by using the FRET mechanism [116].

In addition to the use of nanomaterials for the development of fluorescent biosensors, other studies have described the fabrication of fluorescent viral nucleic acid biosensors on functional patterned chips using nanotechnologies such as lithography, as well as 3D and wax printing techniques [117,118]. Among the various technologies used for the fabrication of fluorescent biosensors, CRISPR/Cas has been intensively studied in recent years [119]. For example, a fluorescent HBV viral DNA biosensor was developed by using CRISPR/Cas12a, MXene nanosheets, and Ag/Pt NPs [120], Moreover, a fluorescent HIV-1 viral DNA biosensor was constructed using CRISPR/Cas12a and hairpin probe-modified magnetic beads [121]. Another study reported a “turn-off” type fluorescent biosensor for the detection of HBV viral DNA using CRISPR/Cas12a, a single-stranded DNA probe, and metal NCs including AuNCs, AgNCs, and CuNCs [122]. In the presence of HBV viral DNA, the CRISPR/Cas12a recognizes the HBV viral DNA and is activated. The activated CRISPR/Cas12a then cleaves the single-stranded DNA probe, thus inhibiting the adsorption of the metal NCs on the single-stranded DNA probe, resulting in the absence of a measurable fluorescent signal (“Turn-off” state). Conversely, in the absence of HBV viral DNA, the CRISPR/Cas12a cannot be activated, and the single-stranded DNA probe is not cleaved. In turn, the metal NCs can adsorb on the single-stranded DNA probe with strong fluorescence emission (“Turn-on” state) (Figure 5C). The developed biosensor detected HBV viral DNA in spiked human sera in a rapid and highly sensitive manner (Picomolar detection sensitivity within 25 min).

In addition to the above-described approaches, a UCNP with unique fluorescence properties has also been used to develop fluorescent viral nucleic acid biosensors [123]. Furthermore, the UCNP-based fluorescent biosensors allow for the facile detection of viruses using portable or compact devices such as smartphones for point-of-care test (POCT) applications. This specific advantage could greatly contribute to the future commercialization of fluorescent viral nucleic acid biosensors [124]. However, fluorescent biosensors require fluorescence detection equipment, which normally is expensive and has a rather large footprint. Moreover, there are still some obstacles that hinder the wider adoption of this technology, such as autofluorescence issues during the detection of targets in real samples such as blood. To overcome these limitations, recent studies on fluorescent biosensors have introduced novel fluorescence nanomaterials such as UCNP that can solve the autofluorescence problem because these probes are excited by NIR wavelengths. Alternatively, other studies have explored the development of fluorescent biosensors that can be operated in the visible light wavelength of mobile phones. The current breakthroughs in nanotechnology and nanomaterials offer a promising basis for the development and commercialization of practical and portable fluorescent viral nucleic acid biosensors, which could prove to be very useful in future disease outbreaks.

## 6. Conclusions and Future Perspectives

Recently, biosensors for the detection of viral nucleic acids have attracted attention in the biomedical field due to the recent global spread of fatal infectious diseases such as the SARS-CoV-2 pandemic in 2020 [89]. The development of a direct method for the detection of disease is particularly important because certain diseases may exhibit a wide variability of symptoms. Several biomarker candidates have been proposed to achieve this, including proteins, viral structures, and nucleic acids. Among these targets, the detection of viral nucleic acid offers key advantages, such as early diagnosis and high accuracy [125]. However, high-performance biosensors are needed to sensitively detect viral nucleic acids. Hence, nanotechnology has recently been employed to develop viral nucleic acid biosensors, and several nanotechnology-assisted biosensors have been reported (Table 1). As described in this review, nanomaterials such as metal oxide, noble metal, and carbon nanomaterials have been widely used as components for biosensors to enhance sensing performance. Moreover, nanotechnologies have been successfully incorporated into biosensors to develop complex sensing platforms.

Despite the promising advances in the field of biosensors in recent years, the detection of viral nucleic acids still requires further improvement. For example, nanostructures must undergo sophisticated optimization prior to their incorporation into biosensors. The shape and size of nanostructures can be diverse and tunable. Moreover, the structural properties of nanomaterials can affect their electrical and optical properties [126]. However, the precise control of nano-scale fabrication methods is extremely difficult, and therefore rough optimization processes are generally implemented [104]. Moreover, the mutation of nucleic acids is an important factor that could affect the sensitivity, accuracy, and reliability of biosensors. Even though some biosensors have been reported to detect mutated viral nucleic acids, additional research is needed to comprehensively identify specific mutation sites and sequences to ensure the accuracy of biosensors [20]. Additionally, to overcome the current issues of biosensors, there must be a close connection between nanotechnologies and biosensor techniques.

Therefore, in this review, we focused on novel nanotechnologies including nanomaterials, nano-fabrication techniques, and other nano-integrated biotechnologies that have been widely applied to develop biosensors for the detection of viral nucleic acids. Given that this review discussed the latest advancements in nanotechnologies, we expect that the findings summarized herein will provide a broad perspective and serve as a foundation for the design and development of future biosensors. Additionally, recently developed biosensors for the detection of viral nucleic acids have incorporated novel detection principles and materials. To improve the sensing performance of these biosensors, a significant amount of research has been focused on the incorporation of different nanotechnologies and novel sensing mechanisms into the development of biosensors. With the integration of nanotechnologies, the development of biosensors was accelerated and elaborated. In addition to accuracy, speed and simplicity are also important factors to consider when developing biosensors. Ideally, biosensors should be operated without the need for complicated sensing systems or sample preparation without compromising accuracy. In turn, this would greatly encourage their wide adoption in the medical field (e.g., POCT applications) [127]. As a platform-based technology, nanotechnology-assisted biosensors could be immediately deployed to aid in future pandemics and other sanitary emergencies. Although this review briefly discussed diagnosis in real samples, we mainly focused on the detection of target nucleic acids with high sensitivity. The sensitivity of biosensors is one of the most important performance indicators in real sample diagnosis. However, given that various substances are present in real samples, the sensitivity and selectivity of biosensors could be negatively affected, and therefore, additional testing is needed to confirm their applicability in the diagnosis of real samples. To this end, nanotechnology has been introduced into the development of biosensors. For instance, the incorporation of UCNPs into the biosensors can diminish the autofluorescence from real blood samples, thus enabling the detection of the targets in real samples with high sensitivity. Future studies should thus focus on evaluating the performance of nanotechnology-based biosensors for the detection of target molecules in real samples. Collectively, the topics discussed in this review provide multiple strategies for the future development of novel biosensors, which could greatly contribute to the early detection of viral diseases and help curb their spread in future disease outbreaks.

## Figures and Tables

**Table 1:** The representative viral nucleic acids biosensors illustrated in this review.

Target	Nanomaterials	Nanotechnology	Detection Method	LoD	Linear Range	Ref.
SARS-CoV-2	Au nanoflower	CRISPR/Cas13a	Electrochemistry	4.4 × 10^−2^ fg/mL (*Orf* gene) 8.1 × 10^−2^ fg/mL (*S* gene)	1.0 × 10^−1^ fg/mL to 1.0 × 10^5^ fg/mL	[94]
	CNTs	-	Electrochemistry	0.33 aM	1 aM to 10 pM	[45]
	Au nanoneedle	4-Wj DNA structure	Electrochemistry	2 copies/μL (*S* gene) 3 copies/μL (*Orf1ab* gene)	100 aM to 10 pM	[89]
	Au nanoporous electrode array	LIL	Electrochemistry	1 fM	100 pM to 1 fM	[20]
	PEI-Ru@Ti_3_C_2_@Au nanoparticles (Mxene)	CRISPR/Cas12a	Electrochemiluminescence	12.8 aM	1 aM- 500 aM	[56]
	CdTe QDs	FRET	Fluorescence	2.52 × nM	-	[116]
	Au nanoparticles	-	SERS	1 fM	1 fM to 1 nM	[18]
	Ag nanoclusters	-	SERS	1 fM (*RdRp* gene) 1 pM (*E* gene)	1 fM to 1 nM (*RdRp* gene) 1 pM to 1 nM (*E* gene)	[103]
Influenza	Ag nanopillars		SERS	41.1 fM	0 pM to 0.159 pM	[38]
HPV	MoS_2_ GO	Nucleic aptamer	Electrochemistry	1.75 pM	3.5 pM to 35.3 pM	[54]
HBV	Mo doped ZnO nanowires	EBL	FET Electrochemistry	1 pM	1 pM to 10 μM	[64]
	CuNCs	CRISPR/Cas12a	Fluorescence	0.54 pM	0.5 pM to 100 pM	[122]
HCV	GQD/AgNC		UV-vis	24.84 pM	25 pM to 500 pM	[42]
	Ag/Zn-MOF	DNA probe	Electrochemistry	0.64 fM	1 fM to 100 nM	[88]
HIV		CRISPR/Cas12a catalytic hairpin DNA assembly	Fluorescence	4.2 fM	10 fM to 100 nM	[121]
HCMV	GO		Fluorescence	68.5 pM	61.0 pM to 488.3 pM	[48]

## Data Availability

Not applicable.

## References

[1] Shrivastav A.M., Cvelbar U., Abdulhalim I. (2021). A comprehensive review on plasmonic-based biosensors used in viral diagnostics. Commun. Biol..

[2] Narita F., Wang Z., Kurita H., Li Z., Shi Y., Jia Y., Soutis C. (2021). A Review of Piezoelectric and Magnetostrictive Biosensor Materials for Detection of COVID-19 and Other Viruses. Adv. Mater..

[3] V’kovski P., Kratzel A., Steiner S., Stalder H., Thiel V. (2021). Coronavirus biology and replication: Implications for SARS-CoV-2. Nat. Rev. Microbiol..

[4] Pastula D.M., Tyler K.L. (2022). An Overview of Monkeypox Virus and Its Neuroinvasive Potential. Ann. Neurol..

[5] Isidro J., Borges V., Pinto M., Sobral D., Santos J.D., Nunes A., Mixão V., Ferreira R., Santos D., Duarte S. (2022). Phylogenomic characterization and signs of microevolution in the 2022 multi-country outbreak of monkeypox virus. Nat. Med..

[6] Baker R.E., Mahmud A.S., Miller I.F., Rajeev M., Rasambainarivo F., Rice B.L., Takahashi S., Tatem A.J., Wagner C.E., Wang L.-F. (2022). Infectious disease in an era of global change. Nat. Rev. Microbiol..

[7] Ilkhani H., Farhad S. (2018). A novel electrochemical DNA biosensor for Ebola virus detection. Anal. Biochem..

[8] Park S., Kim H., Woo K., Kim J.-M., Jo H.-J., Jeong Y., Lee K.H. (2022). SARS-CoV-2 Variant Screening Using a Virus-Receptor-Based Electrical Biosensor. Nano Lett..

[9] Nidzworski D., Siuzdak K., Niedziałkowski P., Bogdanowicz R., Sobaszek M., Ryl J., Weiher P., Sawczak M., Wnuk E., Goddard W.A. (2017). A rapid-response ultrasensitive biosensor for influenza virus detection using antibody modified boron-doped diamond. Sci. Rep..

[10] Courtney S.J., Stromberg Z.R., Kubicek-Sutherland J.Z. (2021). Nucleic Acid-Based Sensing Techniques for Diagnostics and Surveillance of Influenza. Biosensors.

[11] Sanjuán R., Domingo-Calap P. (2016). Mechanisms of viral mutation. Cell. Mol. Life Sci..

[12] Ozer T., Henry C.S. (2021). Paper-based analytical devices for virus detection: Recent strategies for current and future pandemics. TrAC Trends Anal. Chem..

[13] Kang T., Lu J., Yu T., Long Y., Liu G. (2022). Advances in nucleic acid amplification techniques (NAATs): COVID-19 point-of-care diagnostics as an example. Biosens. Bioelectron..

[14] Choi J.R. (2020). Development of Point-of-Care Biosensors for COVID-19. Front. Chem..

[15] Lee E., Lee M., Kwon S., Kim J., Kwon Y. (2022). Systematic and mechanistic analysis of AuNP-induced nanotoxicity for risk assessment of nanomedicine. Nano Converg..

[16] Park J., Kim T.-H., Kwon O., Ismail M., Mahata C., Kim Y., Kim S., Kim S. (2022). Implementation of convolutional neural network and 8-bit reservoir computing in CMOS compatible VRRAM. Nano Energy.

[17] Yoon J., Shin M., Kim D., Lim J., Kim H.-W., Kang T., Choi J.-W. (2022). Bionanohybrid composed of metalloprotein/DNA/MoS2/peptides to control the intracellular redox states of living cells and its applicability as a cell-based biomemory device. Biosens. Bioelectron..

[18] Yin B., Ho W.K.H., Zhang Q., Li C., Huang Y., Yan J., Yang H., Hao J., Wong S.H.D., Yang M. (2022). Magnetic-Responsive Surface-Enhanced Raman Scattering Platform with Tunable Hot Spot for Ultrasensitive Virus Nucleic Acid Detection. ACS Appl. Mater. Interfaces.

[19] Maddali H., Miles C.E., Kohn J., O’Carroll D.M. (2021). Optical Biosensors for Virus Detection: Prospects for SARS-CoV-2/COVID-19. ChemBioChem.

[20] Yoon J., Conley B.M., Shin M., Choi J.-H., Bektas C.K., Choi J.-W., Lee K.-B. (2022). Ultrasensitive Electrochemical Detection of Mutated Viral RNAs with Single-Nucleotide Resolution Using a Nanoporous Electrode Array (NPEA). ACS Nano.

[21] Arunadevi N. (2022). Metal nanocomposites for advanced futuristic biosensing applications. Mater. Lett..

[22] Fritea L., Banica F., Costea T.O., Moldovan L., Dobjanschi L., Muresan M., Cavalu S. (2021). Metal Nanoparticles and Carbon-Based Nanomaterials for Improved Performances of Electrochemical (Bio)Sensors with Biomedical Applications. Materials.

[23] Fujiwara H., Yamauchi K., Wada T., Ishihara H., Sasaki K. (2021). Optical selection and sorting of nanoparticles according to quantum mechanical properties. Sci. Adv..

[24] Cava R., de Leon N., Xie W. (2021). Introduction: Quantum Materials. Chem. Rev..

[25] Pore O.C., Fulari A.V., Velhal N.B., Parale V.G., Park H.H., Shejwal R.V., Fulari V.J., Lohar G.M. (2021). Hydrothermally synthesized urchinlike NiO nanostructures for supercapacitor and nonenzymatic glucose biosensing application. Mater. Sci. Semicond. Process..

[26] Reddy Y.V.M., Shin J.H., Hwang J., Kweon D.-H., Choi C.-H., Park K., Kim S.-K., Madhavi G., Yi H., Park J.P. (2022). Fine-tuning of MXene-nickel oxide-reduced graphene oxide nanocomposite bioelectrode: Sensor for the detection of influenza virus and viral protein. Biosens. Bioelectron..

[27] Sehit E., Altintas Z. (2020). Significance of nanomaterials in electrochemical glucose sensors: An updated review (2016-2020). Biosens. Bioelectron..

[28] Wang Q., Zhang Z., Zhang L., Liu Y., Xie L., Ge S., Yu J. (2022). Photoswitchable CRISPR/Cas12a-Amplified and Co3O4@Au Nanoemitter Based Triple-Amplified Diagnostic Electrochemiluminescence Biosensor for Detection of miRNA-141. ACS Appl. Mater. Interfaces.

[29] Wu Z.-L., Li C.-K., Yu J.-G., Chen X.-Q. (2017). MnO2/reduced graphene oxide nanoribbons: Facile hydrothermal preparation and their application in amperometric detection of hydrogen peroxide. Sens. Actuators B Chem..

[30] Rao D., Zhang X., Sheng Q., Zheng J. (2016). Highly improved sensing of dopamine by using glassy carbon electrode modified with MnO2, graphene oxide, carbon nanotubes and gold nanoparticles. Microchim. Acta.

[31] Choi H.K., Lee M.-J., Lee S.N., Kim T.-H., Oh B.-K. (2021). Noble Metal Nanomaterial-Based Biosensors for Electrochemical and Optical Detection of Viruses Causing Respiratory Illnesses. Front. Chem..

[32] Yang Y., Shu Y., Wu Y., Gao Q. (2021). Co-tuning composition and channel-rich structure of Ag-Pd alloys toward sensitive electrochemical biosensing. Chem. Eng. J..

[33] Maduraiveeran G., Sasidharan M., Ganesan V. (2018). Electrochemical sensor and biosensor platforms based on advanced nanomaterials for biological and biomedical applications. Biosens. Bioelectron..

[34] Wang J., Zhou H., Liu J., He J., Liu J., Yang W. (2021). Electrochemical detection of DNA by formation of efficient electron transfer pathways through adsorbing gold nanoparticles to DNA modified electrodes. Microchem. J..

[35] Wang D., Hua H., Liu Y., Tang H., Li Y. (2019). Single Ag Nanowire Electrodes and Single Pt@Ag Nanowire Electrodes: Fabrication, Electrocatalysis, and Surface-Enhanced Raman Scattering Applications. Anal. Chem..

[36] Yaraki M.T., Tan Y.N. (2020). Metal Nanoparticles-Enhanced Biosensors: Synthesis, Design and Applications in Fluorescence Enhancement and Surface-enhanced Raman Scattering. Chem. Asian J..

[37] Elahi N., Kamali M., Baghersad M.H. (2018). Recent biomedical applications of gold nanoparticles: A review. Talanta.

[38] Park J., Kim J., Park C., Lim J.-W., Yeom M., Song D., Kim E., Haam S. (2022). A flap endonuclease 1-assisted universal viral nucleic acid sensing system using surface-enhanced Raman scattering. Analyst.

[39] Xie P., Yuan W., Liu X., Peng Y., Yin Y., Li Y., Wu Z. (2021). Advanced carbon nanomaterials for state-of-the-art flexible supercapacitors. Energy Storage Mater..

[40] Speranza G. (2021). Carbon Nanomaterials: Synthesis, Functionalization and Sensing Applications. Nanomaterials.

[41] Xu J., Tao J., Su L., Wang J., Jiao T. (2021). A Critical Review of Carbon Quantum Dots: From Synthesis toward Applications in Electrochemical Biosensors for the Determination of a Depression-Related Neurotransmitter. Materials.

[42] Li Y., Li J., Cao Y., Jiang P., Tang Y., Chen Z., Han K. (2022). A visual method for determination of hepatitis C virus RNAs based on a 3D nanocomposite prepared from graphene quantum dots. Anal. Chim. Acta.

[43] Ferrier D.C., Honeychurch K.C. (2021). Carbon Nanotube (CNT)-Based Biosensors. Biosensors.

[44] Rao R., Pint C.L., Islam A.E., Weatherup R.S., Hofmann S., Meshot E.R., Wu F., Zhou C., Dee N., Amama P.B. (2018). Carbon Nanotubes and Related Nanomaterials: Critical Advances and Challenges for Synthesis toward Mainstream Commercial Applications. ACS Nano.

[45] Mujica M.L., Tamborelli A., Castellaro A., Barcudi D., Rubianes M.D., Rodríguez M.C., Saka H.A., Bocco J.L., Dalmasso P.R., Rivas G.A. (2022). Impedimetric and amperometric genosensors for the highly sensitive quantification of SARS-CoV-2 nucleic acid using an avidin-functionalized multi-walled carbon nanotubes biocapture platform. Biosens. Bioelectron. X.

[46] Afroj S., Britnell L., Hasan T., Andreeva D.V., Novoselov K.S., Karim N. (2021). Graphene-Based Technologies for Tackling COVID-19 and Future Pandemics. Adv. Funct. Mater..

[47] Yim Y., Shin H., Ahn S.M., Min D.-H. (2021). Graphene oxide-based fluorescent biosensors and their biomedical applications in diagnosis and drug discovery. Chem. Comm..

[48] Hwang M.T., Heiranian M., Kim Y., You S., Leem J., Taqieddin A., Jing Y., Park I., Van Der Zande A.M., Nam S. (2020). Ultrasensitive detection of nucleic acids using deformed graphene channel field effect biosensors. Nat. Commun..

[49] Lee J.-S., Kim S., Kim S., Ahn K., Min D.-H. (2021). Fluorometric Viral miRNA Nanosensor for Diagnosis of Productive (Lytic) Human Cytomegalovirus Infection in Living Cells. ACS Sensors.

[50] Liu X., He C., Huang Q., Yu M., Qiu Z., Cheng H., Yang Y., Hao X., Wang X. (2022). A facile visualized solid-phase detection of virus-specific nucleic acid sequences through an upconversion activated linear luminescence recovery process. Analyst.

[51] Gogotsi Y., Anasori B. (2019). The Rise of MXenes. ACS Nano.

[52] Zhu J.-H., Feng Y.-G., Wang A.-J., Mei L.-P., Luo X., Feng J.-J. (2021). A signal-on photoelectrochemical aptasensor for chloramphenicol assay based on 3D self-supporting AgI/Ag/BiOI Z-scheme heterojunction arrays. Biosens. Bioelectron..

[53] Rao C.N.R., Gopalakrishnan K., Maitra U. (2015). Comparative Study of Potential Applications of Graphene, MoS2, and Other Two-Dimensional Materials in Energy Devices, Sensors, and Related Areas. ACS Appl. Mater. Interfaces.

[54] Chekin F., Bagga K., Subramanian P., Jijie R., Singh S.K., Kurungot S., Boukherroub R., Szunerits S. (2018). Nucleic aptamer modified porous reduced graphene oxide/MoS2 based electrodes for viral detection: Application to human papillomavirus (HPV). Sens. Actuators B Chem..

[55] Zhang K., Fan Z., Yao B., Ding Y., Zhao J., Xie M., Pan J. (2021). Exploring the trans-cleavage activity of CRISPR-Cas12a for the development of a Mxene based electrochemiluminescence biosensor for the detection of Siglec-5. Biosens. Bioelectron..

[56] Zhang K., Fan Z., Huang Y., Ding Y., Xie M. (2022). A strategy combining 3D-DNA Walker and CRISPR-Cas12a trans-cleavage activity applied to MXene based electrochemiluminescent sensor for SARS-CoV-2 RdRp gene detection. Talanta.

[57] Hatamluyi B., Rezayi M., Amel Jamehdar S., Rizi K.S., Mojarrad M., Meshkat Z., Choobin H., Soleimanpour S., Boroushaki M.T. (2022). Sensitive and specific clinically diagnosis of SARS-CoV-2 employing a novel biosensor based on boron nitride quantum dots/flower-like gold nanostructures signal amplification. Biosens. Bioelectron..

[58] John A., Benny L., Cherian A.R., Narahari S.Y., Varghese A., Hegde G. (2021). Electrochemical sensors using conducting polymer/noble metal nanoparticle nanocomposites for the detection of various analytes: A review. J. Nanostruct. Chem..

[59] Kanwar R., Rathee J., Salunke D.B., Mehta S.K. (2019). Green Nanotechnology-Driven Drug Delivery Assemblies. ACS Omega.

[60] Wu C.-Y., Hsieh H., Lee Y.-C. (2019). Contact Photolithography at Sub-Micrometer Scale Using a Soft Photomask. Micromachines.

[61] Fruncillo S., Su X., Liu H., Wong L.S. (2021). Lithographic Processes for the Scalable Fabrication of Micro- and Nanostructures for Biochips and Biosensors. ACS Sensors.

[62] Li R., Zhao Y., Fan H., Chen M., Hu W., Zhang Q., Jin M., Liu G.L., Huang L. (2022). Versatile nanorobot hand biosensor for specific capture and ultrasensitive quantification of viral nanoparticles. Mater. Today Bio.

[63] Kurt H., Pishva P., Pehlivan Z.S., Arsoy E.G., Saleem Q., Bayazıt M.K., Yüce M. (2021). Nanoplasmonic biosensors: Theory, structure, design, and review of recent applications. Anal. Chim. Acta.

[64] Shariati M., Sadeghi M., Shojaei S.H.R. (2022). Sensory analysis of hepatitis B virus DNA for medicinal clinical diagnostics based on molybdenum doped ZnO nanowires field effect transistor biosensor; a comparative study to PCR test results. Anal. Chim. Acta.

[65] Leitis A., Tseng M.L., John-Herpin A., Kivshar Y.S., Altug H. (2021). Wafer-Scale Functional Metasurfaces for Mid-Infrared Photonics and Biosensing. Adv. Mater..

[66] Liu J., Chen P., Hu X., Huang L., Geng Z., Xu H., Hu W., Wang L., Wu P., Liu G.L. (2023). An ultra-sensitive and specific nanoplasmonic-enhanced isothermal amplification platform for the ultrafast point-of-care testing of SARS-CoV-2. Chem. Eng. J..

[67] Gootenberg J.S., Abudayyeh O.O., Kellner M.J., Joung J., Collins J.J., Zhang F. (2018). Multiplexed and portable nucleic acid detection platform with Cas13, Cas12a, and Csm6. Science.

[68] Kaminski M.M., Abudayyeh O.O., Gootenberg J.S., Zhang F., Collins J.J. (2021). CRISPR-based diagnostics. Nat. Biomed. Eng..

[69] Li S.-Y., Cheng Q.-X., Wang J.-M., Li X.-Y., Zhang Z.-L., Gao S., Cao R.-B., Zhao G.-P., Wang J. (2018). CRISPR-Cas12a-assisted nucleic acid detection. Cell Discov..

[70] Patchsung M., Jantarug K., Pattama A., Aphicho K., Suraritdechachai S., Meesawat P., Sappakhaw K., Leelahakorn N., Ruenkam T., Wongsatit T. (2020). Clinical validation of a Cas13-based assay for the detection of SARS-CoV-2 RNA. Nat. Biomed. Eng..

[71] Fu X., Shi Y., Peng F., Zhou M., Yin Y., Tan Y., Chen M., Yin X., Ke G., Zhang X.-B. (2021). Exploring the Trans-Cleavage Activity of CRISPR/Cas12a on Gold Nanoparticles for Stable and Sensitive Biosensing. Anal. Chem..

[72] Aquino-Jarquin G. (2021). Recent progress on rapid SARS-CoV-2/COVID-19 detection by CRISPR-Cas13-based platforms. Drug Discov. Today.

[73] Xiao M., Lai W., Man T., Chang B., Li L., Chandrasekaran A.R., Pei H. (2019). Rationally engineered nucleic acid architectures for biosensing applications. Chem. Rev..

[74] Ma L., Liu J. (2020). Catalytic Nucleic Acids: Biochemistry, Chemical Biology, Biosensors, and Nanotechnology. iScience.

[75] Shen L., Wang P., Ke Y. (2021). DNA Nanotechnology-Based Biosensors and Therapeutics. Adv. Healthc. Mater..

[76] Guo X., Chen L., Li P., Li X., Wang J., Guo L., Yang H. (2022). Construction of electrochemiluminescence biosensor via click chemistry and ARGET-ATRP for detecting tobacco mosaic virus RNA. Anal. Biochem..

[77] Kong D., Wang X., Gu C., Guo M., Wang Y., Ai Z., Zhang S., Chen Y., Liu W., Wu Y. (2021). Direct SARS-CoV-2 Nucleic Acid Detection by Y-Shaped DNA Dual-Probe Transistor Assay. J. Am. Chem. Soc..

[78] Yang H., Zhou Y., Liu J. (2020). G-quadruplex DNA for construction of biosensors. TrAC Trends Anal. Chem..

[79] Bialy R.M., Mainguy A., Li Y., Brennan J.D. (2022). Functional nucleic acid biosensors utilizing rolling circle amplification. Chem. Soc. Rev..

[80] Wu N., Zhang H.-C., Sun X.-H., Guo F.-N., Feng L.-X., Yang T., Wang J.-H. (2022). Detection of HIV/HCV virus DNA with homogeneous DNA machine-triggered in situ formation of silver nanoclusters. Sens. Actuators B Chem..

[81] Thévenot D.R., Toth K., Durst R.A., Wilson G.S. (2001). Electrochemical biosensors: Recommended definitions and classification. Biosens. Bioelectron..

[82] Diculescu V.C., Chiorcea-Paquim A.-M., Oliveira-Brett A.M. (2016). Applications of a DNA-electrochemical biosensor. TrAC Trends Anal. Chem..

[83] Islam M.N., Masud M.K., Haque M.H., Hossain M.S.A., Yamauchi Y., Nguyen N.-T., Shiddiky M.J.A. (2017). RNA Biomarkers: Diagnostic and Prognostic Potentials and Recent Developments of Electrochemical Biosensors. Small Methods.

[84] Erdem A., Eksin E., Congur G. (2015). Indicator-free electrochemical biosensor for microRNA detection based on carbon nanofibers modified screen printed electrodes. J. Electroanal. Chem..

[85] Cho I.-H., Kim D.H., Park S. (2020). Electrochemical biosensors: Perspective on functional nanomaterials for on-site analysis. Biomater. Res..

[86] Chowdhury A.D., Takemura K., Li T.-C., Suzuki T., Park E.Y. (2019). Electrical pulse-induced electrochemical biosensor for hepatitis E virus detection. Nat. Commun..

[87] Cajigas S., Alzate D., Orozco J. (2020). Gold nanoparticle/DNA-based nanobioconjugate for electrochemical detection of Zika virus. Microchim. Acta.

[88] El-Sheikh S.M., Osman D.I., Ali O.I., Shousha W.G., Shoeib M.A., Shawky S.M., Sheta S.M. (2021). A novel Ag/Zn bimetallic MOF as a superior sensitive biosensing platform for HCV-RNA electrochemical detection. Appl. Surf. Sci..

[89] Kashefi-Kheyrabadi L., Nguyen H.V., Go A., Baek C., Jang N., Lee J.M., Cho N.-H., Min J., Lee M.-H. (2022). Rapid, multiplexed, and nucleic acid amplification-free detection of SARS-CoV-2 RNA using an electrochemical biosensor. Biosens. Bioelectron..

[90] Ji D., Guo M., Wu Y., Liu W., Luo S., Wang X., Kang H., Chen Y., Dai C., Kong D. (2022). Electrochemical Detection of a Few Copies of Unamplified SARS-CoV-2 Nucleic Acids by a Self-Actuated Molecular System. J. Am. Chem. Soc..

[91] Moço A.C.R., Neto J.A.S., de Moraes D.D., Guedes P.H., Brussasco J.G., Flauzino J.M.R., Luz L.F.G., Soares M.M.C.N., Madurro J.M., Brito-Madurro A.G. (2021). Carbon ink-based electrodes modified with nanocomposite as a platform for electrochemical detection of HIV RNA. Microchem. J..

[92] Najjar D., Rainbow J., Timilsina S.S., Jolly P., de Puig H., Yafia M., Durr N., Sallum H., Alter G., Li J.Z. (2022). A lab-on-a-chip for the concurrent electrochemical detection of SARS-CoV-2 RNA and anti-SARS-CoV-2 antibodies in saliva and plasma. Nat. Biomed. Eng..

[93] Lee Y., Choi J., Han H.-K., Park S., Park S.Y., Park C., Baek C., Lee T., Min J. (2021). Fabrication of ultrasensitive electrochemical biosensor for dengue fever viral RNA Based on CRISPR/Cpf1 reaction. Sens. Actuators B Chem..

[94] Heo W., Lee K., Park S., Hyun K.-A., Jung H.-I. (2022). Electrochemical biosensor for nucleic acid amplification-free and sensitive detection of severe acute respiratory syndrome coronavirus 2 (SARS-CoV-2) RNA via CRISPR/Cas13a trans-cleavage reaction. Biosens. Bioelectron..

[95] Bukkitgar S.D., Shetti N.P., Aminabhavi T.M. (2021). Electrochemical investigations for COVID-19 detection-A comparison with other viral detection methods. Chem. Eng. J..

[96] Tanwar S., Paidi S.K., Prasad R., Pandey R., Barman I. (2021). Advancing Raman spectroscopy from research to clinic: Translational potential and challenges. Spectrochim. Acta A Mol. Biomol. Spectrosc..

[97] Serebrennikova K.V., Berlina A.N., Sotnikov D.V., Zherdev A.V., Dzantiev B.B. (2021). Raman Scattering-Based Biosensing: New Prospects and Opportunities. Biosensors.

[98] Shipp D.W., Sinjab F., Notingher I. (2017). Raman spectroscopy: Techniques and applications in the life sciences. Adv. Opt. Photon..

[99] Wu L., Dias A., Diéguez L. (2022). Surface enhanced Raman spectroscopy for tumor nucleic acid: Towards cancer diagnosis and precision medicine. Biosens. Bioelectron..

[100] Cao Y., Sun M. (2022). Tip-enhanced Raman spectroscopy. Rev. Phys..

[101] Kneipp K., Wang Y., Kneipp H., Perelman L.T., Itzkan I., Dasari R.R., Feld M.S. (1997). Single Molecule Detection Using Surface-Enhanced Raman Scattering (SERS). Phys. Rev. Lett..

[102] Zhang Y.-J., Chen S., Radjenovic P., Bodappa N., Zhang H., Yang Z.-L., Tian Z.-Q., Li J.-F. (2019). Probing the Location of 3D Hot Spots in Gold Nanoparticle Films Using Surface-Enhanced Raman Spectroscopy. Anal. Chem..

[103] Jang A.S., Kumar P.P.P., Lim D.-K. (2022). Attomolar Sensitive Magnetic Microparticles and a Surface-Enhanced Raman Scattering-Based Assay for Detecting SARS-CoV-2 Nucleic Acid Targets. ACS Appl. Mater. Interfaces.

[104] Choi J.-H., Shin M., Yang L., Conley B., Yoon J., Lee S.-N., Lee K.-B., Choi J.-W. (2021). Clustered Regularly Interspaced Short Palindromic Repeats-Mediated Amplification-Free Detection of Viral DNAs Using Surface-Enhanced Raman Spectroscopy-Active Nanoarray. ACS Nano.

[105] Su A., Liu Y., Cao X., Xu W., Liang C., Xu S. (2022). A universal CRISPR/Cas12a-mediated AuNPs aggregation-based surface-enhanced Raman scattering (CRISPR/Cas-SERS) platform for virus gene detection. Sens. Actuators B Chem..

[106] Yin B., Zhang Q., Xia X., Li C., Ho W.K.H., Yan J., Huang Y., Wu H., Wang P., Yi C. (2022). A CRISPR-Cas12a integrated SERS nanoplatform with chimeric DNA/RNA hairpin guide for ultrasensitive nucleic acid detection. Theranostics.

[107] Zhang J., Miao X., Song C., Chen N., Xiong J., Gan H., Ni J., Zhu Y., Cheng K., Wang L. (2022). Non-enzymatic signal amplification-powered point-of-care SERS sensor for rapid and ultra-sensitive assay of SARS-CoV-2 RNA. Biosens. Bioelectron..

[108] Ma F., Li Y., Tang B., Zhang C.-y. (2016). Fluorescent Biosensors Based on Single-Molecule Counting. Acc. Chem. Res..

[109] Zhou H., Zhang S. (2022). Recent Development of Fluorescent Light-Up RNA Aptamers. Crit. Rev. Anal. Chem..

[110] Ryoo S.-R., Lee J., Yeo J., Na H.-K., Kim Y.-K., Jang H., Lee J.H., Han S.W., Lee Y., Kim V.N. (2013). Quantitative and Multiplexed MicroRNA Sensing in Living Cells Based on Peptide Nucleic Acid and Nano Graphene Oxide (PANGO). ACS Nano.

[111] Choi J.-H., Lim J., Shin M., Paek S.-H., Choi J.-W. (2021). CRISPR-Cas12a-Based Nucleic Acid Amplification-Free DNA Biosensor via Au Nanoparticle-Assisted Metal-Enhanced Fluorescence and Colorimetric Analysis. Nano Lett..

[112] Hu R., Liu T., Zhang X.-B., Huan S.-Y., Wu C., Fu T., Tan W. (2014). Multicolor Fluorescent Biosensor for Multiplexed Detection of DNA. Anal. Chim. Acta.

[113] Xu Z., Chen D., Li T., Yan J., He T., Hu R., Li Y., Yang Y., Liu M. (2022). Microfluidic space coding for multiplexed nucleic acid detection via CRISPR-Cas12a and recombinase polymerase amplification. Nat. Commun..

[114] Choi J.-H., Ha T., Shin M., Lee S.-N., Choi J.-W. (2021). Nanomaterial-Based Fluorescence Resonance Energy Transfer (FRET) and Metal-Enhanced Fluorescence (MEF) to Detect Nucleic Acid in Cancer Diagnosis. Biomedicines.

[115] Li D., Chen H., Gao X., Mei X., Yang L. (2021). Development of General Methods for Detection of Virus by Engineering Fluorescent Silver Nanoclusters. ACS Sensors.

[116] Bardajee G.R., Zamani M., Sharifi M. (2021). Efficient and Versatile Application of Fluorescence DNA-Conjugated CdTe Quantum Dots Nanoprobe for Detection of a Specific Target DNA of SARS Cov-2 Virus. Langmuir.

[117] Wei S.-C., Chang C.-C., Chuang T.-L., Sung K.-B., Lin C.-W. (2022). Rapid Detection of Virus Nucleic Acid via Isothermal Amplification on Plasmonic Enhanced Digitizing Biosensor. Biosensors.

[118] Teengam P., Nisab N., Chuaypen N., Tangkijvanich P., Vilaivan T., Chailapakul O. (2021). Fluorescent paper-based DNA sensor using pyrrolidinyl peptide nucleic acids for hepatitis C virus detection. Biosens. Bioelectron..

[119] Huang Z., Liu S., Pei X., Li S., He Y., Tong Y., Liu G. (2022). Fluorescence Signal-Readout of CRISPR/Cas Biosensors for Nucleic Acid Detection. Biosensors.

[120] Tao Y., Yi K., Wang H., Kim H.-W., Li K., Zhu X., Li M. (2022). CRISPR-Cas12a-regulated DNA adsorption and metallization on MXenes as enhanced enzyme mimics for sensitive colorimetric detection of hepatitis B virus DNA. J. Colloid Interface Sci..

[121] Zhao X., Tian X., Wang Y., Li L., Yu Y., Zhao S., Zhang J. (2022). CRISPR-Cas12a-activated palindrome-catalytic hairpin assembly for ultrasensitive fluorescence detection of HIV-1 DNA. Anal. Chim. Acta.

[122] Tao Y., Yi K., Wang H., Li K., Li M. (2022). Metal nanoclusters combined with CRISPR-Cas12a for hepatitis B virus DNA detection. Sens. Actuators B Chem..

[123] Alexaki K., Kyriazi M.E., Greening J., Taemaitree L., El-Sagheer A.H., Brown T., Zhang X., Muskens O.L., Kanaras A.G. (2022). A SARS-Cov-2 sensor based on upconversion nanoparticles and graphene oxide. RSC Adv..

[124] Huang C.-H., Park Y.I., Lin H.-Y., Pathania D., Park K.S., Avila-Wallace M., Castro C.M., Weissleder R., Lee H. (2019). Compact and Filter-Free Luminescence Biosensor for Mobile in Vitro Diagnoses. ACS Nano.

[125] Zou M., Su F., Zhang R., Jiang X., Xiao H., Yan X., Yang C., Fan X., Wu G. (2021). Rapid point-of-care testing for SARS-CoV-2 virus nucleic acid detection by an isothermal and nonenzymatic Signal amplification system coupled with a lateral flow immunoassay strip. Sens. Actuators B Chem..

[126] Kim J.-M., Lee C., Lee Y., Lee J., Park S.-J., Park S., Nam J.-M. (2021). Synthesis, Assembly, Optical Properties, and Sensing Applications of Plasmonic Gap Nanostructures. Adv. Mater..

[127] Wang L., Wang X., Wu Y., Guo M., Gu C., Dai C., Kong D., Wang Y., Zhang C., Qu D. (2022). Rapid and ultrasensitive electromechanical detection of ions, biomolecules and SARS-CoV-2 RNA in unamplified samples. Nat. Biomed. Eng..

